# *In-situ* construction and repair of SEI via a Li_2_ZrF_6_ additive for durable lithium metal batteries

**DOI:** 10.1093/nsr/nwag385

**Published:** 2026-06-23

**Authors:** Bohao Peng, Tao Wang, Ewa Mijowska, Xuecheng Chen, Yuping Wu

**Affiliations:** Confucius Energy Storage Lab, School of Energy and Environment & Z Energy Storage Center, Southeast University, China; Confucius Energy Storage Lab, School of Energy and Environment & Z Energy Storage Center, Southeast University, China; Faculty of Chemical Technology and Engineering, West Pomeranian University of Technology in Szczecin, Poland; Center for Advanced Materials and Manufacturing Process Engineering (CAMMPE), West Pomeranian University of Technology in Szczecin, Poland; Faculty of Chemical Technology and Engineering, West Pomeranian University of Technology in Szczecin, Poland; Center for Advanced Materials and Manufacturing Process Engineering (CAMMPE), West Pomeranian University of Technology in Szczecin, Poland; Confucius Energy Storage Lab, School of Energy and Environment & Z Energy Storage Center, Southeast University, China

**Figure 1.**
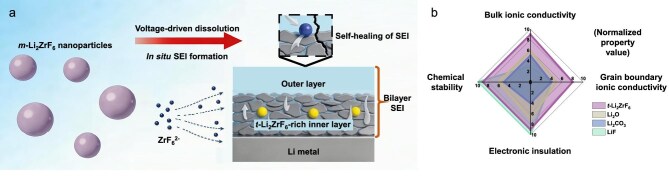
The dynamic precursor strategy for SEI repair for LMBs. (a) Schematic illustration of the mechanism of the *m*-Li_2_ZrF_6_. (b) Performance comparison of *t*-Li_2_ZrF_6_ with other inorganic SEI components (adapted with permission from Springer Nature).

The quest to surpass the energy density ceiling of conventional lithium-ion batteries has placed lithium (Li) metal anodes at the forefront of next-generation energy storage [1,2]. However, the commercial viability of Li metal batteries is severely compromised by the inherent fragility of the solid electrolyte interphase (SEI). Traditional SEI layers, formed spontaneously via electrolyte reduction, are static. Under the colossal volume fluctuations of Li plating and stripping, these passive layers fracture, failing to self-heal and leading to rampant dendrite growth. To address this, a recent landmark study in *Nature* by Xu and co-workers introduced an ingenious dynamic precursor strategy utilizing monoclinic Li_2_ZrF_6_ (*m*-Li_2_ZrF_6_) nanoparticles as an electrolyte additive [3]. This approach abandons static protection in favor of a voltage-driven dissolution mechanism (Fig. 1a): under operational voltage, *m*-Li_2_ZrF_6_ releases ZrF_6_^2−^, which migrate to the anode surface and chemically convert into a bilayer SEI rich in trigonal Li_2_ZrF_6_ (*t*-Li_2_ZrF_6_).

From a materials perspective, *t*-Li_2_ZrF_6_ exhibits exceptional physicochemical properties ideal for SEI construction (Fig. 1b) [4]. First-principles calculations reveal that *t*-Li_2_ZrF_6_ possesses an ultra-low Li^+^ migration barrier, significantly outperforming conventional SEI components like LiF, ensuring rapid ion transport. Crucially, its wide band gap and chemical stability can effectively prevent the continuous decomposition of the electrolyte. Furthermore, the strong adsorption energy of Li on the *t*-Li_2_ZrF_6_ surface homogenizes nucleation.

Mechanistically, the advantage of this strategy lies in its dynamic voltage‑driven dissolution process, distinct from conventional additives. During battery operation, the nanoparticles dissolve under voltage drive, releasing ZrF_6_^2−^. These ions migrate to the Li metal surface, where they undergo spontaneous chemical reactions (rather than electrochemical reduction) to *in situ* form an SEI inner layer rich in *t*-Li_2_ZrF_6_. Cryogenic transmission electron microscopy and other characterizations confirm that this SEI exhibits a unique bilayer architecture—an inner crystalline layer composed of *t*-Li_2_ZrF_6_ and Li_2_O, and an outer layer containing organic products from electrolyte decomposition. This strategy also demonstrates dynamic self‑healing capability. When the SEI is damaged during cycling, ZrF_6_^2−^ migrates to the defective sites and regenerates *t*-Li_2_ZrF_6_  *in-situ*, achieving real-time repair. Specifically, the Li||LFP cell exhibited an initial coulombic efficiency of 97.65% and maintained 80% capacity retention after 3000 cycles at 1C with a specific capacity of 115.1 mAh g^−1^. The system incorporating *m*-Li_2_ZrF_6_ additive during cycling exhibits uniform and dense Li deposition along with low electrolyte consumption, markedly outperforming conventional systems.

Polymer-based coatings for Li metal accommodate volumetric changes via mechanical buffering and viscoelasticity, but increase interfacial impedance and suffer from electrolyte-induced swelling. Dual-additive systems enable the synergistic optimization of impurity scavenging, flame retardancy, SEI, and the cathode electrolyte interphase (CEI) formation, thereby overcoming the limitations of single additives. However, they are constrained by a narrow dosing window, where excess amounts can induce adverse effects. Sacrificial additives rapidly optimize initial interfaces through preferential reactions but lack sustained regeneration, compromising long-term stability. In contrast, the Li_2_ZrF_6_ strategy combines long-cycle life and high-rate performance while offering inherent self-healing capabilities.

The true impact of this dynamic precursor strategy may extend far beyond Li metal anodes. The fundamental principles could provide transformative insights for the broader field of interfacial engineering in other high-capacity electrode systems plagued by unstable SEI, such as Si, Na, and K. However, to bridge the gap between proof-of-concept and practical implementation, several critical challenges must be decisively addressed.

First, the universality of this approach requires deeper interrogation. While *m*-Li_2_ZrF_6_ proves highly effective in low-voltage Li||LFP cells, its performance drastically deteriorates in high-voltage Li||NCM811 configurations. This decline cannot be simplistically attributed to transition-metal dissolution alone. The elevated voltage may trigger excessive ZrF_6_^2−^ release, inducing unforeseen parasitic reactions within both the SEI and the CEI. Systematically decoupling these intertwined degradation pathways is paramount. Mitigating transition metal dissolution in NCM811 requires multifaceted strategies, including particle size optimization, elemental doping, and electrolyte engineering. Notably, employing NCM811 with low specific surface area, doping with elements like Al and Mg, applying protective surface coatings (e.g. MgO, LiNbO_3_), and leveraging electrolyte additives to form a robust CEI have been proved highly effective. Furthermore, the exploration of other transition‑metal fluorides (Li_*x*_MF_*y*_) remains a fertile ground. Leveraging AI-driven high-throughput computational screening to map material genomes for optimal ionic conductivity, electronic insulation, and dissolution kinetics will undoubtedly accelerate the discovery of next-generation, cost-effective dynamic precursors.

Second, stability during long‑term cycling warrants rigorous examination. An ideal SEI must exhibit not only high ionic conductivity and low electronic conductivity, but also viscoelastic properties capable of accommodating the extreme volume fluctuations of the Li anode without fracturing. The microscopic deformation and fracture mechanics of the *t*-Li_2_ZrF_6_-based SEI under realistic stress remain poorly understood. Concurrently, the amount of *m*-Li_2_ZrF_6_ added is limited and cannot indefinitely repair the SEI. The rate of SEI damage during deep cycling will eventually outpace the supply of healing ZrF_6_^2−^. Establishing a quantitative correlation between additive concentration, SEI failure rates, and cell lifespan is critically needed to optimize electrolyte formulations.

Finally, scaling this technology from laboratory to industrial manufacturing presents formidable engineering hurdles. Ensuring the formation of a stable, homogeneous *m*-Li_2_ZrF_6_ nanoparticle suspension without aggregation or sedimentation requires concerted efforts in materials engineering. Specifically, the aggregation of nanoparticles can be prevented by introducing functional groups onto the particle surface, adsorbing surfactants, incorporating dispersants that provide electrostatic repulsion, and employing ultrasonic dispersion and high-shear mixing processes. Moreover, the *in situ* synthesis of nanoparticles in the electrolyte is expected to further address the engineering issues related to large-scale production. A systematic evaluation of the rheological properties and long-term colloidal stability of these composite electrolytes will be indispensable for their successful deployment in commercial battery manufacturing.

In summary, although the dynamic‑precursor strategy is conceptually advanced and promising, its universality, stability, and the engineering challenges associated with scalable production remain critical issues that must be addressed before practical implementation.
